# Current perspectives on the training of Oral Pathology specialists in Brazil: a cross-sectional study

**DOI:** 10.4317/medoral.27059

**Published:** 2025-08-16

**Authors:** Luiz Miguel Ferreira, Samuel Trezena, João Pedro Santos Nascimento, Marcos Paulo Maia-Lima, Paulo Rogério Ferreti Bonan, Sabina Pena Borges Pêgo, Hercílio Martelli-Júnior

**Affiliations:** 1 Department of Oral Diagnosis, Piracicaba Dental School, State University of Campinas (FOP-UNICAMP), Piracicaba, São Paulo, Brazil; 2 Postgraduate Program in Health Sciences, State University of Montes Claros (UNIMONTES), Montes Claros, Minas Gerais, Brazil; 3 Community Public Health and Epidemiology, School of Dentistry, State University of Montes Claros (UNIMONTES), Montes Claros, Minas Gerais, Brazil; 4 Postgraduate Program in Dentistry, Federal University of Paraíba (UFPB), João Pessoa, Brazil; 5 Oral Pathology and Oral Medicine, Dental School, State University of Montes Claros (UNIMONTES), Montes Claros, Minas Gerais, Brazil

## Abstract

**Background:**

Oral Pathology (OP) is an important part of diagnosing and managing oral and maxillofacial diseases. Despite being recognized as a specialty in Brazil for over 50 years, significant gaps remain in the availability of specialists and training programs. Therefore, this study aims to map and analyze the training of specialists in OP in Brazil.

**Material and Methods:**

A cross-sectional study was conducted using publicly available data from Brazilian governmental databases, including the Federal Council of Dentistry, the Brazilian Institute of Geography and Statistics, and the Ministry of Education. Variables analyzed included the number of OP specialists, their geographic distribution, training opportunities, and population coverage. Descriptive and comparative analyses were performed using Microsoft® Excel (version 2410) and Statistical Package for Social Sciences® (version 27.0).

**Results:**

In 2024, 424 active OP specialists were registered in Brazil, composed of 240 women and 184 men, representing only 0.3% of all dental specialists. The ratio of OP specialists to inhabitants was 1:478,964, with marked regional disparities. The Southeast had the highest density, while the North had the lowest, with some states lacking any OP specialists. Additionally, 12 active OP training programs were identified, primarily concentrated in the Southeast. Most programs were distance learning, limiting opportunities for practical training. Temporal analysis revealed a decline in OP specialist registrations over the past two decades, despite population growth.

**Conclusions:**

Brazil faces challenges in OP training, including insufficient specialists, uneven regional distribution and limited hands-on training opportunities. The expansion of hybrid training models and the promotion of the OP specialty among dental students are vital to address these issues. Collaborative efforts between educational institutions, professional organizations, and the government are essential to strengthen the specialty and improve early diagnosis rates of oral cancer.

** Key words:**Oral pathology, teaching, specialization, education, dental, graduate.

## Introduction

Oral Pathology (OP) is an important area of specialization within the healthcare system (1), and along with Oral Medicine (OM) is essential for diagnosis and treatment of oral and maxillofacial complex diseases. In Brazil, OP specialists are involved not only in histological diagnosis, but also in clinical care services, providing assistance, treatments and performing biopsies (2,3). According to the Brazilian Federal Council of Dentistry (Conselho Federal de Odontologia - CFO, in Portuguese), the OP specialty focuses on studying histological aspects and utilizing laboratory, microscopic, and biochemical resources for the diagnosis and prognosis of conditions. Additionally, OP professionals are responsible for conducting and interpreting various laboratory tests and requisitioning complementary examinations to aid in diagnosing pathologies of the stomatognathic system (https://website.cfo.org.br/).

In Brazil the OP specialty was recognized before that of OM. The recognition of OP as a formal specialty has a long history. The country was the first in Latin America to officially register OP (2,3), constituting a significant milestone in the development of the field. A pioneering milestone in the history of the formation of the OP in Brazil started as an intensive course taught by Professor William G. Shafer at the Federal University of Rio de Janeiro, in one month, to fourteen professors from different Brazilian states (4). However, even after 50 years since OP was recognized as a specialty in Brazil, there are still significant gaps in the availability of specialist training courses.

At the same time, epidemiological studies have demonstrated a significant increase in oral, oropharyngeal, and tongue cancer incidence over the past three decades. While this trend is primarily associated with established risk factors, timely diagnosis remains challenging due to complex healthcare system barriers (5-8). The diagnostic process involves multiple stages, where general dental practitioners play a crucial role in initial detection, while oral pathologists provide essential histopathological confirmation. Current limitations in specialist availability, working hours, and laboratory infrastructure may contribute to diagnostic delays, though their impact on overall cancer incidence should not be overstated (9,10).

A recent study (11) has raised great discussions regarding the training of professionals in this area in Brazil. The findings highlight an insufficient number of OP specialists, even considering that over the course of a 35-year career, a general dentist is likely to diagnose an average of two to three patients with oral cancer (OC) and 675 patients with oral potentially malignant disorders. These statistics underscore the urgent need to increase both the number and preparedness of specialists to improve early diagnosis rates. Thus, the present study aims to map and analyze the training of specialists in OP in Brazil by examining specific data around specialization courses and registered specialists.

## Material and Methods

- Study Design

This report was written following the criteria present on the Strengthening the Reporting of Observational Studies in Epidemiology (STROBE) guideline for cross-sectional studies (12). This research is a cross-sectional study analyzing publicly accessible data from Brazilian governmental databases, which does not require approval from an ethics committee as it involves only secondary data that is publicly available and does not include identifiable personal information.

- Data collection and availability

All data were collected between August 25th and September 13th, 2024. Data were obtained from the official websites of the CFO (https://website.cfo.org.br/dados-estatisticos-de-profissionais-e-entidades-ativas-por-especialidade/) and the Brazilian Institute of Geography and Statistics (Instituto Brasileiro de Geografia e Estatística - IBGE, in Portuguese) (https://censo2022.ibge.gov.br/panorama/). The CFO registry provided the number of OP specialists with active registrations, including gender and registration year. Population data was sourced from the 2022 census by IBGE to calculate the ratio of OP specialists per capita by region. Data on current specialization courses in OP were gathered from the Ministry of Education (Ministério da Educação - MEC, in Portuguese) through the e-MEC portal (https://emec.mec.gov.br/emec/nova), access was made on September 1st, 2024. Search was conducted with the “advanced search” tool, the keywords used were: “patologia oral” (oral pathology), “patologia bucal” (oral pathology) and “patologia oral e maxilofacial” (oral and maxillofacial pathology). Both the “in-person” and “online” modalities were selected, as well as the “active courses” and “inactive courses” categories. After the searches, spreadsheets were downloaded directly from the portal and unified for analysis. Duplicated courses were removed.

This data includes course modality, distribution by region, and total annual openings, providing a basis for evaluating the availability and accessibility of OP training across different regions of Brazil. Data were collected by two independent and blind authors and cross-checked later.

- Eligibility Criteria

Data from OP specialists with active licenses in Brazil and data from active specialization courses in OP were included in this study. Inactive professionals or courses were manually excluded from the extracted sheets.

- Variables

The variables considered for this study were: the number of OP specialists registered in the CFO register, the year of registration, sex, region and state of the professionals. The number of specialization courses in OP in the MEC register, its modalities, workload, state and number of openings registered. And finally, the number of inhabitants in all Brazilian states and regions.

- Statistical Analysis

Descriptive statistics were used to characterize the distribution of OP specialists by sex, geographic region, and population coverage. The availability of OP specialization courses, both active and inactive, was also described, considering geographic region, number of vacancies, and course workload. The ratios of OP specialists per 100,000 inhabitants were calculated for each state and region to illustrate the disparities in access to specialized OP services. Microsoft® Excel 2016 (version 2410) (Microsoft Corp., Redmond, WA, USA) and Statistical Package for the Social Sciences (SPSS)® (version 27.0) (IBM Corp., Armonk, NY, USA) were used to perform these calculations, producing Tables and graphical representations to facilitate comparisons. The data were also analyzed in terms of temporal trends, focusing on registration patterns over the past two decades and on regional disparities.

## Results

In August 2024, there were 424 registered OP specialists with active licenses in Brazil. This group, composed of 240 women and 184 men, represented approximately 0.3% of the 143,341 dental specialists registered nationwide. Given Brazil’s population of 203,080,756 inhabitants (2022 census), this translates to an average of 478,964 inhabitants per OP specialist, highlighting a limited availability of OP professionals relative to the general population.

**Figure 1:**
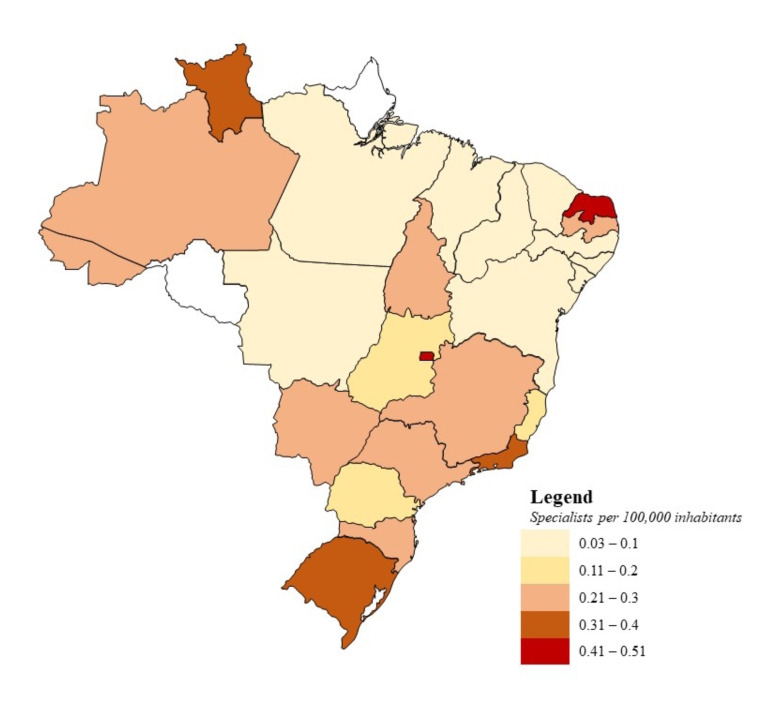
The average number of Oral Pathology specialists per 100,000 inhabitants

The regional distribution of OP specialists demonstrated significant disparities. The Southeast concentrated the largest number of specialists [242], followed by the South [72], Northeast [55], Midwest [30], and North [25], illustrating a marked imbalance in specialist availability across Brazil’s extensive territories. States such as Amapá and Rondônia had no registered OP specialists (Table 1). Fig. 1 presents the ratio of OP specialists per 100,000 inhabitants, with a national average of 0.2 specialists. The highest density of OP specialists was observed in Rio Grande do Norte (0.51 specialists per 100,000 inhabitants), followed by the Federal District [0.42] and Rio de Janeiro [0.33]. In contrast, states such as Piauí [0.03], Bahia [0.04], and Ceará [0.05] had the lowest indicators, highlighting a professional shortage in certain areas of the country.

**Figure 2:**
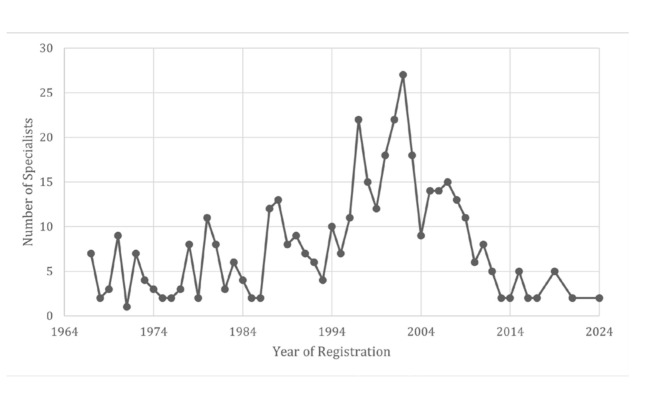
Number of Oral Pathology specialists and the year the specialty was registered in the Federal Council of Dentistry (CFO).

A more detailed temporal analysis revealed that the first specialists in OP were registered in the profession in 1967, totaling seven professionals, which accounted for 1.7% of the total number of specialists. By comparison, two OP professionals (0.5%) were newly registered in 2024. The highest number of OP specialist registrations was recorded in 2002, with 27 new registrations (6.4%) (Fig. 2). The graph shows that, over the past 20 years, the number of specialists has progressively decreased, in contrast to the country's population growth and the number of dentists.

**Figure 3:**
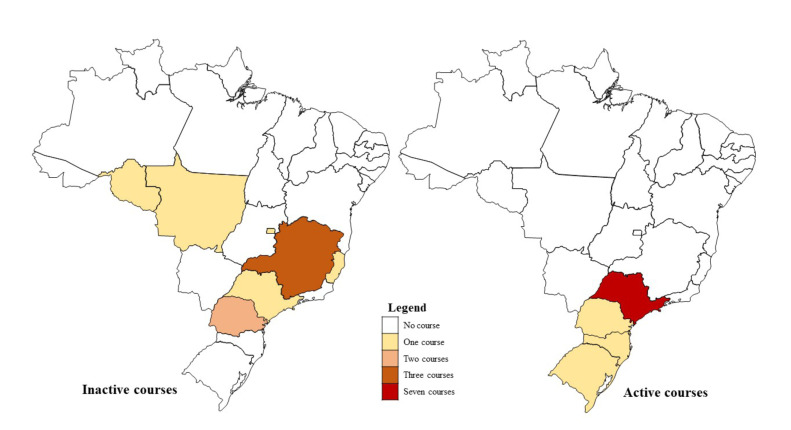
Geographical distribution of specialization courses in Oral Pathology in Brazil, according to activity status in September 2024.

Twenty OP training programs were identified, of which eight (40%) were inactive and offered 2,240 vacancies. Most of the inactive programs were offered in face-to-face mode (75%) and the workload varied from 480 to 905 hours. The inactive programs were offered in the states of Paraná, Rondônia, Mato Grosso, Espírito Santo, Distrito Federal, Minas Gerais and São Paulo. It is important to note that one of the courses is delivered through educational hubs located across Minas Gerais, São Paulo, and the Federal District. For this reason, each hub was considered individually in the analysis (Fig. 3).

The concentration of active OP training programs varied across regions, with 12 active programs identified, primarily concentrated in the Southeast. Three of these programs were offered in face-to-face mode (25%), while the remainder were distance-learning courses. São Paulo had the highest number of program offerings, while other states in the South and Southeast accounted for the remainder. The specialization courses had a workload ranging from 600 to 1,100 hours with a total of 3,298 vacancies offered annually (Table 2).

## Discussion

This study reveals a critical shortage and uneven distribution of OP specialists in Brazil, with only 424 active professionals serving 203 million inhabitants (1:478,964 ratio). The Southeast concentrates 57% of specialists, while entire states (e.g., Amapá, Rondônia) remain unserved. Stark regional disparities exist in specialist density (0.51-0.03/100,000 inhabitants). Alarmingly, new specialist registrations have declined since 2002 (peaking at *n*=27) to just two in September 2024, contrasting with population growth. Of 20 training programs identified, 40% were inactive, predominantly face-to-face courses in underserved regions, while active programs (*n*=12) were mostly distance-learning and concentrated in developed areas. These findings suggest both a contracting workforce and systemic training inadequacies exacerbating geographic inequities.

The low number of specialists identified is similar to the reality in other Latin American countries. In a previous study (2) that analyzed the proportions between the number of specialists and the general population in South American countries and Mexico, a large variation was identified. In Paraguay, there is only one specialist in OP, while in Peru and Venezuela, the proportion is one for more than 4.5 million inhabitants. In Brazil, the proportion is one for 479,000 inhabitants. Although the number of specialists is still low in Brazil, the country stands out considering its territorial and population size (3).

This specialist scarcity extends beyond Latin America. In Taiwan (population approximately 22 million), only 70 oral pathologists exist, with fewer than 10% performing histopathological diagnoses (13). Similarly, Arab Middle Eastern countries report fewer than 10 specialists in some countries, with some nations lacking any recorded OP professionals or reliable workforce data (1). While Brazil’s ratio (1:479,000) remains inadequate, it outperforms several global counterparts. These disparities highlight a transnational crisis in OP healthcare workforce development, exacerbated by uneven training infrastructure and likely compounded by the same educational disincentives observed in dental education systems worldwide.

The examination of the available specialization training in OP reveals a concentration of programs in certain regions of Brazil, particularly in São Paulo and the South and Southeast regions. This concentration highlights a significant disparity in access to specialized education, particularly for dental professionals in the North and Northeast regions, where opportunities for specialized training are more limited (14,15). This concentration of educational institutions in the Southeast may help to understand why two states in the North have no specialists. Even though a considerable number of positions are available, it is important to note that not all of them are filled, as the majority are distance learning, which reflects the shortage of OP specialists in the current scenario.

Although a considerable number of specialization courses are offered online, which can mitigate disparities related to access regardless of geographic location, it is interesting to highlight the importance of practical training in the diagnosis and management of oral diseases (16). Practical experience is crucial for developing clinical skills, particularly in OP, where precise diagnostic and management abilities are vital, especially in cases of OC. In addition, in Brazil, the required workload to become a specialist recognized by the CFO in OP is 500 hours, with a minimum of 10% theoretical classes and 80% practical classes (https://website.cfo.org.br/). Consequently, offering a fully online course is unfeasible, as professionals would not be able to register the specialty with the professional council.

In alignment with the expansion of distance learning in OP training (representing 75% of active programs), telepathology has emerged as a strategic complementary tool to address access barriers and specialist shortages. Particularly valuable when employed by professionals with hybrid training backgrounds, this approach enhances diagnostic capabilities through remote case discussion and interprofessional collaboration. The COVID-19 pandemic demonstrated telepathology's potential to create vital support networks among specialists across distant locations (17,18) - a particularly relevant solution for Latin American contexts marked by both high clinical demands and resource limitations. When integrated with existing distance education frameworks in OP, telepathology can help bridge the gap created by geographic maldistribution of specialists while maintaining diagnostic quality standards.

Regarding the Stricto sensu postgraduate program (master’s and PhD) in Brazil, there is only one program dedicated specifically to OP. The other programs offer this specialty only as an area of research concentration, all focused exclusively on academic training. In a previous study, we analyzed the professional trajectory of 1,220 graduates from postgraduate programs in OP and OM in Brazil, who graduated between 2013 and 2021. We observed that most work as professors (540; 44.3%) and are involved in academic and research activities, without working directly with laboratory diagnostics (19). No master's or PhD programs with a concentration in OP focused on the professional (clinical practice) area were identified (https://sucupira.capes.gov.br/). In addition, since June 2nd 2010, the CFO no longer recognizes diplomas issued by master’s or PhD programs as the criteria for specialist registration (https://sistemas.cfo.org.br/visualizar/atos/DECIS%C3%83O/SEC/2010/36). This change in CFO legislation may help explain the decline in registrations over the past decade.

The global scarcity of OP specialists appears rooted in declining student interest, as evidenced by international studies showing minimal career pursuit intentions [16% in India (20) and 94.3% rejection rates in Taiwan (21)]. While Brazil lacks specific studies on this phenomenon, its critically low specialist-to-population ratio strongly mirrors these trends. This disinterest likely stems from educational challenges, including difficulties in undergraduate OP comprehension (22) and uninspiring clinical experiences, which collectively deter specialization choices (23). Addressing this crisis requires curricular restructuring to enhance accessibility and appeal of OP training. However, the absence of national studies on Brazilian students' perceptions represents a critical knowledge gap in developing effective recruitment strategies for this dwindling workforce.

The low demand for an OP career may be associated with financial compensation. Studies suggest that the choice of this specialty is often influenced by the preference for surgical or clinical practice over academia and that the possibility of private practice could increase interest in the field (24,25). However, in Brazil, few OP specialists work exclusively in private practice due to the low spontaneous demand for their services, making other dental specialties financially more attractive. Additionally, wages in the public sector are relatively low, which may further discourage professionals from pursuing a career in this field. It is important to note the scarcity of scientific literature specifically addressing the economic factors influencing the choice of OP in Brazil.

A limitation of this study was the exclusive use of secondary data from public databases, which may restrict a detailed analysis of aspects such as the quality of training programs and the practical work of specialists. In particular, the study relied solely on the e-MEC website, as it is a public and accessible source that consolidates information on courses offered by institutions accredited by the MEC, in accordance with CFO Resolution No. 74 of July 20, 2007. Additionally, the fee charged by the CFO for specialty registration may discourage professionals from completing the process, contributing to an underreporting of the actual number of specialists. Given these limitations, future studies are needed to investigate the training pathways, students' experiences during specialization, and the impacts of their education on clinical and academic practice.

In conclusion, the training of OP specialists in Brazil faces significant challenges, including a shortage of professionals, regional disparities, and the limitations of online education. Expanding in-person and hybrid programs, as well as promoting the OP specialty among dental students are essential steps to ensure that the country is better equipped to meet the growing demand for OP services. Collaborative efforts between educational institutions, professional organizations, and the government will be crucial in addressing these challenges and fostering the growth of this specialty.

## Figures and Tables

**Table 1:** Distribution of Oral Pathology specialists per inhabitant in Brazilian regions and states in September 2024.

Region	State	Inhabitants (n)	Specialists (n) (%)	Specialists per Inhabitants
North	Acre	830,018	2 (0.5)	1:415,009
	Amapá	733,759	0	-
	Amazonas	3,941,613	12 (2.8)	1:328,467.75
	Pará	8,120,131	5 (1.8)	1:1,624,026.20
	Rondônia	1,581,196	0	-
	Roraima	636,707	2 (0.5)	1:318,353.50
	Tocantins	1,511,460	4 (1.0)	1:377,865
Northeast	Alagoas	3,127,683	3 (0.3)	1:1,042,531
	Bahia	14,141,626	6 (1.5)	1:2,356,937.67
	Ceará	8,794,957	5 (1.2)	1:1,758,991.40
	Maranhão	6,776,699	5 (1.2)	1:1,355,339.80
	Paraíba	3,974,687	9 (2.1)	1:441,631.89
	Pernambuco	9,058,931	8 (1.9)	1:1,132,366.38
	Piauí	3,271,199	1 (0.2)	1:3,271,199
	Rio Grande do Norte	3,302,729	17 (4.0)	1:194,278.18
	Sergipe	2,210,004	1 (0.2)	1:2,210,004
Southeast	Espírito Santo	3,833,712	6 (1.5)	1:638,952
	Minas Gerais	20,539,989	49 (11.5)	1:419,183.45
	Rio de Janeiro	16,055,174	54 (12.7)	1:297,318.04
	São Paulo	44,411,238	133 (31.5)	1:333,919.08
South	Paraná	11,444,380	21 (4.9)	1:544,970.48
	Rio Grande do Sul	10,882,965	34 (8.0)	1:320,087.21
	Santa Catarina	7,610,361	17 (4.0)	1:447,668.29
Midwest	Distrito Federal	2,817,381	12 (2.8)	1:234,781.75
	Goiás	7,056,495	9 (2.1)	1:784,055
	Mato Grosso	3,658,649	3 (0.3)	1:1,219,549.67
	Mato Grosso do Sul	2,757,013	6 (1.5)	1:459,502.17

**Table 2:** Active Specialization Courses in Oral Pathology in Brazil, according to the Ministry of Education, as of September 2024, through the e-MEC platform.

Course Title	Affiliation	Modality	Workload	State	Region	n. of Openings
Oral Pathology Studies	UniBF Centro Universitário	Distance	1100	Paraná	South	20
Oral and Maxillofacial Pathology	Faculdade SOBRESP de Santa Maria	Distance	600	Rio Grande do Sul	South	50
Oral and Maxillofacial Pathology	Centro Universitário Avantis	Face-to-face	885	Santa Catarina	South	12
Oral and Maxillofacial Pathology	Faculdade Metropolitana do Estado de São Paulo	Distance	600	São Paulo	Southeast	500
Oral and Maxillofacial Pathology	Faculdade Metropolitana de Franca	Distance	855	São Paulo	Southeast	500
Oral and Maxillofacial Pathology	Faculdade Metropolitana do Estado de São Paulo	Distance	855	São Paulo	Southeast	500
Buccal/Oral Pathology	Faculdade São Leopoldo Mandic	Face-to-face	681	São Paulo	Southeast	12
Buccal/Oral Pathology	Universidade Federal do Rio de Janeiro	Face-to-face	735	Rio de Janeiro	Southeast	4
Buccal/Oral Pathology	Faculdade Metropolitana do Estado de São Paulo	Distance	600	São Paulo	Southeast	500
Buccal/Oral Pathology	Faculdade Metropolitana de Franca	Distance	600	São Paulo	Southeast	500
Buccal/Oral Pathology	Faculdade Unyleya	Distance	360	Rio de Janeiro	Southeast	200
Buccal/Oral Pathology	Minas Faculdade	Distance	700	São Paulo	Southeast	500

Legend: UniBF - União Brasileira de Faculdades; SOBRESP - Sociedade Brasileira para o Ensino e Pesquisa.
